# Factors influencing the attainment of major motor milestones in CDKL5 deficiency disorder

**DOI:** 10.1038/s41431-022-01163-1

**Published:** 2022-08-18

**Authors:** Kingsley Wong, Mohammed Junaid, Scott Demarest, Jacinta Saldaris, Tim A. Benke, Eric D. Marsh, Jenny Downs, Helen Leonard

**Affiliations:** 1 Telethon Kids Institute, The University of Western Australia, Perth, WA Australia; 2 Children’s Hospital Colorado, Pediatric Neurology, University of Colorado School of Medicine, Aurora, USA; 3 Division of Neurology, Children’s Hospital of Philadelphia, School of Medicine, University of Pennsylvania, Philadelphia, USA

**Keywords:** Genetics research, Paediatrics

## Abstract

This study investigated the influence of factors at birth and in infancy on the likelihood of achieving major motor milestones in CDKL5 Deficiency Disorder (CDD). Data on 350 individuals with a pathogenic *CDKL5* variant was sourced from the International CDKL5 Disorder Database. A first model included factors available at birth (e.g., sex, variant group and mosaicism) and the second additionally included factors available during infancy (e.g., age at seizure onset, number of anti-seizure medications used, experience of a honeymoon period and formal therapy). Cox regression was used to model the time to achieve the milestones. The probability of attaining the outcomes at specific ages was estimated by evaluating the time-to-event function at specific covariate values. Independent sitting and walking were achieved by 177/350 and 57/325 children respectively. By seven years of age, 67.1% of females but only 37.3% of males could sit independently. About a quarter each of females and males achieved independent walking by eight and six years, respectively. When observed from birth, female gender, a late truncating variant and mosaicism impacted most positively on the likelihood of independent sitting. When observed from one year, later seizure onset and experiencing a honeymoon period also improved the likelihood of independent sitting. Factors that favoured sitting (except gender) also improved walking. Having a truncating variant between aa178 and aa781 reduced the likelihood of achieving independent sitting and walking. It is possible to utilise factors occurring early in life to inform the likelihood of future motor development in CDD.

## Introduction

CDKL5 Deficiency Disorder (CDD) is a relatively newly recognised Developmental Epileptic Encephalopathy, originally thought to be an atypical form of Rett syndrome with which it shares many characteristics [1–3]. However, it is the early onset seizures, later becoming refractory, and the severe motor, cognitive [2] and cortical visual [4, 5] impairment that are the main differentiating clinical features. CDD is caused by a pathogenic variant in the *CDKL5* gene which encodes for CDKL5, a serine threonine kinase, with a role in the regulation of axon outgrowth, dendritic morphogenesis and synapse formation early in life [6]. Recent data has indicated that over 200 different CDKL5 variants are harboured by as few as 285 affected individuals making it challenging to assess genotype phenotype relationships [7]. Thus, many studies have categorised variants into four groups according to their position on the gene and functional consequences [8].

The extreme rarity of this disorder [9, 10] prompted the need for an international database, the International CDKL5 Disorder Database (ICDD), with capacity to accrue a large enough number of cases to understand the clinical profile of this disorder and the variability therein [11, 12]. The first published study using these data (*n* = 127) found that by the age of five years less than three quarters of females and less than half of males had achieved independent sitting [12]. The ability to walk was even further compromised with only a quarter of females able to walk by the age of four and a half years and only one of 18 males achieving this ability. Depending on their family’s socioeconomic status and the resources available in their country of residence it would be expected that many children with CDD may be provided with early intervention therapies at a young age. However, there is no published data to confirm this and thus no evaluation of the benefits or otherwise.

Onset of seizures early in life is generally the main presentation in CDD with six weeks the median age of onset and the majority commencing in the first year of life [13]. These seizures tend to be difficult to control and can require polypharmacy, with its own inherent adverse effects, from an early age. However just under half of affected children do experience a honeymoon period, i.e. a brief period of seizure control [14, 15], when seizures appear to remit for a median duration of six months but with a range of 2 months to 11 years [4, 13].

There is variability in the attainment of milestones in CDD. For example, some females may achieve independent sitting by age 6 months whilst one quarter would still not have achieved this milestone by five years of age [12]. Similar variability has been described with the development of walking in females. Males, on the other hand, tend to be more delayed except if they are mosaic [7].

Increasingly infants are being diagnosed with CDD due to expanding use of epilepsy gene panels [16], but at present, there is no way to predict their developmental outcomes. With higher seizure burden at an earlier time point potentially associated with subsequent poorer development [17], the question emerges as to whether developmental trajectories are predetermined at birth or if factors occurring in the first year of life including effectiveness of seizure treatment or access to early intervention may alter the trajectory.

The aims of this study were to use available data from the ICDD to estimate the likelihood of children with CDD achieving the major milestones of sitting and walking by certain time points, and to investigate the impact of influencing factors for which data were available including child’s sex, variant group, age at seizure onset, occurrence of a honeymoon period, number of anti-seizure medications used and receipt of formal therapy in the first year of life.

## Methods

### Data source and study population

The International CDKL5 Disorder Database (ICDD) served as the data source for this study [12]. We included children who had a pathogenic or likely pathogenic *CDKL5* variant and whose families completed a baseline questionnaire between 2012 and 2021. Data was supplemented when available by information from a follow-up questionnaire completed between 2018 and 2019.

### Outcome variables

The outcomes variables for this study were age at independent sitting and independent walking. Independent sitting and walking related to the times at first reported episode of sitting and walking without support, respectively. Outcome data were mostly drawn from the baseline questionnaire. However, we also updated these details from the follow-up questionnaire if information related to independent sitting and/or walking had previously been missing or the child had not reached these milestones at the time of submission of the baseline questionnaire.

### Explanatory variables

#### Variant group and mosaicism

As previously done [7, 8], variants were grouped into the following categories: 1. no functional protein, comprising any variant that prevents function in the catalytic domain; 2. missense/in-frame variants within the catalytic domain, including any missense variant within the protein kinase active region or in-frame variant that results in loss of some kinase region (with consequent protein intact) due to a deletion; 3. truncating variants occurring between amino acid (aa) 172 and aa781, including any truncating variant such as a nonsense or frameshift variant that causes loss of C- terminal region whilst maintaining kinase activity; and 4. late truncating variants occurring after aa781 including those that maintain the kinase activity and a large portion of the C-terminal region. Those individuals whose variants could not be grouped in either of the above-mentioned categories were classified as “other variants”. Mosaicism was considered to be present when indicated on the genetic report.

#### Age at seizure onset

Age at seizure onset was defined as the age of the child (in months) when the first seizure episode was observed or reported by parents/caregivers. For analysis the variable was grouped into two categories “≤1.5 months” and “>1.5 months”.

#### Number of anti-seizure medications prescribed in the first year of life

We dichotomised the number of anti-seizure medications (ASMs) used in the first year of life as either up to three medications or four or more per day.

#### Ever honeymoon period

In our study a honeymoon period was considered to be a seizure-free episode of >2 months after seizure onset. Data on ever experiencing a honeymoon period was dichotomised into two groups “Yes” and “No”.

#### Formal therapy during the first year of life

Formal therapy referred to those children who had undergone either physiotherapy or occupational therapy during their first year of life. This variable was categorised as either “Yes” or “No”.

### Statistical analyses

Descriptive statistics were used to summarise the characteristics of the study population. For time-to-event analysis, all individuals were followed from birth until censoring (i.e. time of independent sitting/walking or date of last questionnaire returned, whichever occurred first). Total observation time was the sum of follow-up time for each individual. Cox proportional hazard regression was used to estimate the likelihood of achieving the milestones using the explanatory variables, and the corresponding beta coefficients, hazard ratios and their 95% confidence intervals were reported. The proportional hazard assumption was tested and confirmed using the Schoenfeld residuals and graphical assessment.

The probability of achieving the milestones at specific age (sitting: 1, 1.5, 2, 3 and 5 years; walking: 1.5, 2, 4, and 6 years) was estimated by evaluating the time-to-event function at specific covariate values of four selected profiles (Supplementary Table 1). For sitting, individuals selected for profile A had truncating variants after aa781, with no mosaicism, experienced a honeymoon period, took three or less anti-seizure medications during the first year of life, had a later seizure onset (>1.5 months), and received formal therapy during their first year of life. Profile B was similar to Profile A except for the presence of mosaicism. Profile C was also similar to profile A except for variant group (missense/in-frame variants) and number of ASMs (≥4). Profile D was selected as having the least favourable outcome and included those individuals with truncating variants occurring between amino acid (aa) 172 and aa781, with no mosaicism, who had never experienced a honeymoon period, took four or more anti-seizure medication in the first year of life, had an earlier seizure onset (≤1.5 months), and did not receive formal therapy during their first year of life. Profiles selected for estimating the probability of independent walking were similar to those selected for sitting except for exclusion of formal therapy in the first year of life for which we felt the time frame was inappropriate. The log-log based confidence interval for the likelihood estimates was derived from the asymptomatic variance of the covariate-adjusted time-to-event function as described by Marubini and Valsecchi [18]. Two models were developed to accommodate explanatory variables. In the first model we included only those covariates that were available at birth (e.g., sex, variant group and mosaicism) and in the second model we additionally included those that became available during first year of life (e.g., age at seizure onset, number of ASMs used in first year of life, experience of a honeymoon period and formal therapy). The starting age of observation was at birth in the first model and at one year of age (1 and 1.5 years for walking) in the second model. In the second model, individuals who were censored prior to the start of observation period were excluded. All analyses were carried out using Stata 16.0 (Stata Corp, College Station, TX, USA).

## Results

Clinical, genetic, and demographic characteristics of the 350 individuals with CDD are presented in Supplementary Table 2. The majority (83.4%) of individuals were female and just under half (46.3%) were born in North America. The median age at most recent questionnaire was 5.7 years (interquartile range [IQR] 2.4,10.9). A quarter (24.9%) had variants classified as no functional protein, 31.7% as missense/in-frame variants within the kinase domain and 27.4% as truncating variants from aa172 to aa781. A smaller proportion (10.6%) had truncating variants after aa781, and mosaicism was reported in the genetic reports of 10 (2.9%) individuals (seven (12%) males and three (1%) females). In just over a half (56.6%) of individuals seizure onset was at or before one and a half months. During their first year of life almost a half (47.7%) were on four or more ASMs, just over a third (34.8%) on two or three ASMs and a small proportion (10.6%) took none or only one ASM. More than half experienced a honeymoon period (56.3%) and nearly two thirds (63.4%) received formal therapy during their first year of life.

### Independent sitting

Independent sitting was achieved by 177 children over a total observation period of 1262.2 years. Close to a quarter (22.3%, 95% confidence interval [CI] 18.2, 27.2) of those who ever learned to sit were able to do so by one year of age. Among females, the median time to sitting was 26 months (95% CI 24, 36) and over two thirds (67.1%, 95% CI 60.6, 73.5) achieved this milestone by seven years. On the other hand, slightly less than a quarter (23.0%, 95% CI 13.7, 37.1) of males were sitting by two years and only 37.5% (95% CI 23.5, 56.0) by seven years. Among the explanatory variables, when observed from birth, being female had the largest impact on the likelihood of independent sitting (hazard ratio [HR] 3.0, 95% CI 1.7, 5.1), followed by presence of mosaicism (HR 2.5, 95% CI 1.1, 5.4) and having a truncating variant after aa781 (HR 1.7, 95% CI 1.0, 3.0) (Table 1). When observed from one year of age (*n* = 242, number of events = 102, total observation time = 935.1 years), being female (HR 3.5, 95% CI 1.6, 7.9), having a truncating variant after aa781 (HR 2.1, 95% CI 1.0, 4.6) and ever experiencing a honeymoon period (HR 1.8, 95% CI 1.1, 2.8) had the largest influence on the chance of achieving the milestone (Table 1). Formal therapy during first year of life (HR 1.36, 95% CI 0.82, 2.25) and taking three or fewer ASMs in the first year of life (HR 1.19, 95% CI 0.76, 1.84) had less influence.

**Table 1:** Multivariable regression analysis of time to independent sitting in individuals with CDKL5 deficiency disorder, by starting age of observation.

Starting age of observation (year)	0	1	0	1
*n*	350	197	350	197
	HR (95% CI)		β (95% CI)	
Sex				
Female	2.97 (1.75, 5.11)	3.52 (1.57, 7.90)	1.09 (0.56, 1.63)	1.26 (0.45, 2.07)
Male	Ref	Ref	Ref	Ref
Variant group				
Truncating variants between aa178 and aa781	Ref	Ref	Ref	Ref
Truncating variants after aa781	1.72 (1.00, 2.95)	2.11 (0.96, 4.62)	0.54 (0.003,1.08)	0.74 (−0.04, 1.53)
No functional protein	1.57 (1.02, 2.39)	1.50 (0.80, 2.79)	0.45 (0.03, 0.87)	0.40 (−0.22, 1.03)
Missense/in-frame	1.37 (0.92, 2.04)	1.57 (0.87, 2.84)	0.31 (−0.09, 0.71)	0.45 (−0.14, 1.04)
Other variants	1.19 (0.58, 2.46)	1.67 (0.56, 4.95)	0.18 (−0.55, 0.90)	0.51 (−0.58, 1.60)
Mosaicism				
Absent	Ref	Ref	Ref	Ref
Present	2.47 (1.14, 5.35)	1.57 (0.50, 4.96)	0.91 (0.13, 1.68)	0.45 (−0.69, 1.60)
Ever honeymoon period				
No	–	Ref	–	Ref
Yes	–	1.78 (1.12, 2.81)	–	0.57 (0.12, 1.03)
Number of ASM used in first year of life				
0–3	–	1.19 (0.76, 1.84)	–	0.17 (−0.27, 0.61)
≥4	–	Ref	–	Ref
Age at onset of seizures (month)				
≤1.5	–	Ref	–	Ref
>1.5	–	1.41 (0.90, 2.22)	–	0.35 (−0.10, 0.80)
Formal therapy during first year of life				
No	–	Ref	–	Ref
Yes	–	1.36 (0.82, 2.25)	–	0.31 (−0.19, 0.81)

*Ref* reference category, *aa* amino acid, *ASM* anti-seizure medication, *HR* hazard ratio.

### Independent walking

Among the 325 individuals with data on independent walking, 57 achieved the milestone over a total observation time of 2,037.6 years. Time-to-event analysis revealed that a quarter each of females and males achieved independent walking by 8 (95% CI 4.5, –) and 6 (95% CI 3.5, –) years, respectively. When observed from birth, ‘truncating variants after aa781’ had the largest impact on the probability of independent walking (HR 2.7, 95% CI 1.2, 6.4), followed by presence of mosaicism (HR 2.5, 95% CI 0.9, 7.1) and ‘other variants’ (HR 2.3, 95% CI 0.8, 6.6) (Table 2). These factors, in addition to ever experiencing a honeymoon period (HR 1.9, 95% CI 1.0, 3.5), were highly ranked when observation started from one year of age (*n* = 290, number of events = 53, total observation time = 1713.6 years). Shifting the starting age of observation to 1.5 years (*n* = 266, number of events = 45, total observation time = 1573.6 years) increased the importance of the honeymoon period (HR 2.5, 95% CI 1.3, 5.2), whilst other factors such as ‘truncating variants after aa781’, ‘other variants’ and presence of mosaicism remained influential (Table 2). In contrast to the findings for independent sitting, gender played a lesser role in affecting the likelihood of independent walking. However, as with sitting, having three or fewer ASMs in the first year of life had minimal influence.

**Table 2:** Multivariable regression analysis of time to independent walking in individuals with CDKL5 Deficiency Disorder, by starting age of observation.

Starting age of observation (year)	0	1	1.5	0	1	1.5
*n*	325	262	240	325	262	240
	HR (95% CI)			β (95% CI)		
Sex						
Female	1.32 (0.63, 2.77)	1.30 (0.56, 3.04)	1.24 (0.49, 3.15)	0.28 (−0.47, 1.02)	0.26 (−0.59, 1.11)	0.22 (−0.72, 1.15)
Male	Ref	Ref	Ref	Ref	Ref	Ref
Variant group						
Truncating variants between aa178 and aa781	Ref	Ref	Ref	Ref	Ref	Ref
Truncating variants after aa781	2.73 (1.17, 6.35)	2.24 (0.92, 5.47)	2.64 (0.96, 7.28)	1.00 (0.16,1.85)	0.80 (−0.09, 1.70)	0.97 (−0.04, 1.98)
No functional protein	1.54 (0.71, 3.32)	1.24 (0.55, 2.81)	1.28 (0.49, 3.32)	0.43 (−0.34, 1.20)	0.21 (−0.60, 1.03)	0.24 (−0.71, 1.20)
Missense/in-frame	0.97 (0.43, 2.18)	0.95 (0.39, 2.34)	1.37 (0.51, 3.69)	−0.03 (−0.84, 0.78)	−0.05 (−0.94, 0.85)	0.31 (−0.04, 1.98)
Other variants	2.29 (0.79, 6.61)	2.11 (0.62, 7.16)	2.17 (0.53, 8.84)	0.83 (−0.23, 1.89)	0.75 (−0.47, 1.97)	0.77 (−0.63, 2.18)
Mosaicism						
Absent	Ref	Ref	Ref	Ref	Ref	Ref
Present	2.51 (0.89, 7.08)	1.87 (0.54, 6.49)	2.21 (0.61, 8.01)	0.92 (−0.12, 1.96)	0.62 (−0.62, 1.87)	0.79 (−0.49,2.08)
Ever honeymoon period						
No	–	Ref	Ref	–	Ref	Ref
Yes	–	1.88 (1.00, 3.51)	2.54 (1.25, 5.18)	–	0.63 (0.003, 1.25)	0.93 (0.22,1.65)
Number of ASM used in first year of life						
0–3	–	1.55 (0.83, 2.90)	1.47 (0.74, 2.94)	–	0.44 (−0.19, 1.06)	0.39 (−0.30,1.08)
≥4	–	Ref	Ref	–	Ref	Ref
Age at seizure onset (month)						
≤1.5	–	Ref	Ref	–	Ref	Ref
>1.5	–	1.68 (0.93, 3.04)	1.68 (0.88, 3.21)	–	0.52 (−0.07, 1.11)	0.52 (−0.13, 1.17)

*Ref* reference category, *aa* amino acid, *ASM* anti-seizure medication, *HR* hazard ratio.

### Likelihood of independent sitting and walking over time

**Fig. 1:**
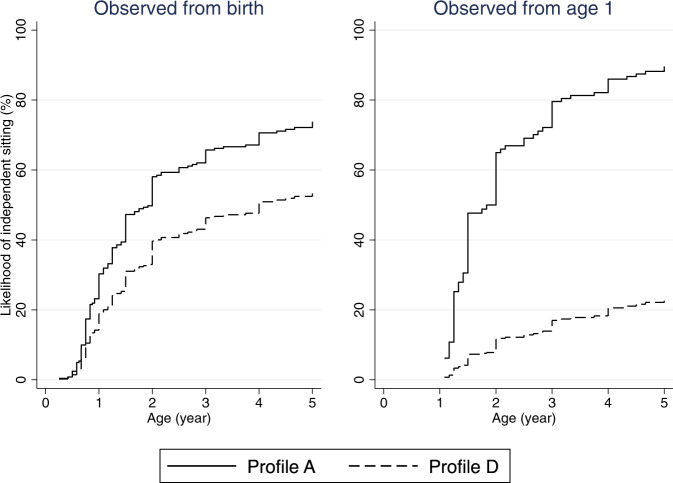
Estimated time to independent sitting curves in females with CDD, by starting age of observation. Profile A: Variant group (truncating variants after aa781), mosaicism (absent), ever honeymoon period (yes), number of anti-seizure medications used in first year of life (0–3), age at seizure onset (>1.5 months), formal therapy during first year of life (yes). Profile D: Variant group (truncating variants between aa172 and aa781), mosaicism (absent), ever honeymoon period (no), number of anti-seizure medications used in first year of life (≥4), age at seizure onset (≤1.5 months), formal therapy during first year of life (no).

**Fig. 2:**
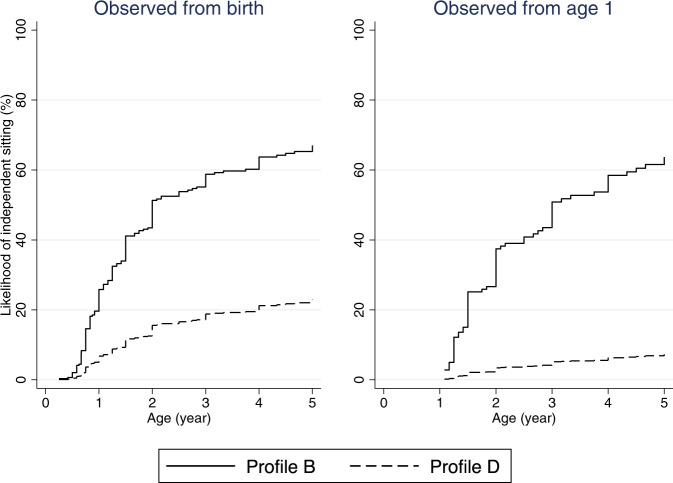
Estimated time to independent sitting curves in males with CDD, by starting age of observation. Profile B: Variant group (truncating variants after aa781), mosaicism (present), ever honeymoon period (yes), number of anti-seizure medications used in first year of life (0–3), age at seizure onset (>1.5 months), formal therapy during first year of life (yes). Profile D: Variant group (truncating variants between aa172 and aa781), mosaicism (absent), ever honeymoon period (no), number of anti-seizure medications used in first year of life (≥4), age at seizure onset (≤1.5 months), formal therapy during first year of life (no).

**Fig. 3:**
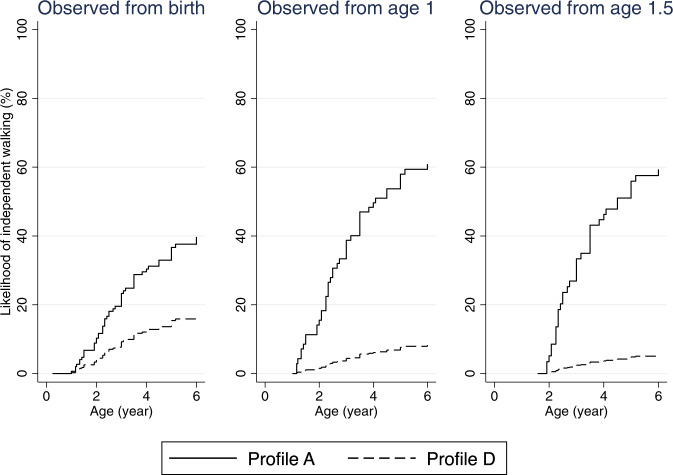
Estimated time to independent walking curves in females with CDD, by starting age of observation. Profile A: Variant group (truncating variants after aa781), mosaicism (absent), ever honeymoon period (yes), number of anti-seizure medications used in first year of life (0–3), age at seizure onset (>1.5 months), formal therapy during first year of life (yes). Profile D: Variant group (truncating variants between aa172 and aa781), mosaicism (absent), ever honeymoon period (no), number of anti-seizure medications used in first year of life (≥4), age at seizure onset (≤1.5 months), formal therapy during first year of life (no).

**Fig. 4:**
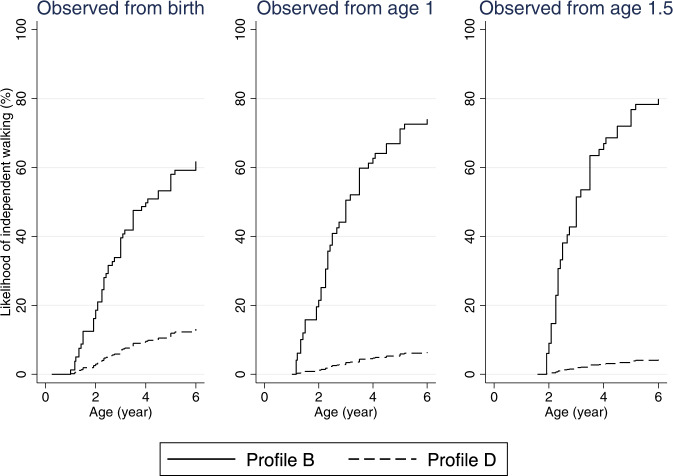
Estimated time to independent walking curves in males with CDD, by starting age of observation. Profile B: Variant group (truncating variants after aa781), mosaicism (present), ever honeymoon period (yes), number of anti-seizure medications used in first year of life (0–3), age at seizure onset (>1.5 months), formal therapy during first year of life (yes). Profile D: Variant group (truncating variants between aa172 and aa781), mosaicism (absent), ever honeymoon period (no), number of anti-seizure medications used in first year of life (≥4), age at seizure onset (≤1.5 months), formal therapy during first year of life (no).

The estimated probability of independent sitting and walking at specific age (by sex, profile of explanatory variables and starting age of observation) is shown in Tables 3, 4 and Figs. 1–4. In general, females were more likely to achieve either milestone than males. Furthermore, the time-to-event curves of a favourable and much less favourable profiles (female: Profile A and Profile D; male: Profile B and Profile D) changed as more pertinent information became available later in life (Figs. 1–4). For instance, as seen in Table 3, when the observation period started at birth, for females with truncating variants after aa781 and without mosaicism (i.e., Profile A – most favourable) the likelihood of independent sitting at 5 years of age was 73.8% (95% CI 57.7, 87.6), compared with 54.1% (95% CI 43.7, 65.3) for those with truncating variants between aa172 and aa781 and without mosaicism (i.e., Profile D – least favourable). When medical history during the first year of life was taken into account and observation started at one year, the likelihood of sitting unaided at 5 years of age in females with Profile A who had experienced a honeymoon period, used less than 4 ASMs, had later seizure onset and received formal therapy was 89.6% (95% CI 65.8, 99.2) (Table 3). In contrast the likelihood of achieving the same was only 23.3% (95% CI 12.8, 40.3) when observed from one year of age for those with Profile D who had truncating variants between aa172 and aa781 and no mosaicism and for whom no honeymoon period had been reported, had used > =4 ASMs), had a history of early seizure onset and received no formal therapy during their first year of life (Table 3).

**Table 3:** Estimated probability of independent sitting at 1, 1.5, 2, 3, and 5 years of age in individuals with CDKL5 deficiency disorder, by sex, profile of explanatory variables and starting age of observation.

Profile	Start age (yr)	Female (age in year)						Male (age in year)					
			Probability % (95% CI)						Probability % (95% CI)				
		*n*	1	1.5	2	3	5	*n*	1	1.5	2	3	5
A	0	292	30.3 (20.3, 43.6)	47.3 (33.6, 63.2)	58.1 (42.9, 74.0)	65.7 (49.8, 81.0)	73.8 (57.7, 87.6)	58	11.4 (6.1, 20.6)	19.3 (10.8, 33.1)	25.3 (14.5, 41.8)	30.1 (17.5, 48.7)	36.2 (21.4, 56.7)
	1	159		47.7 (26.2, 74.9)	65.0 (39.7, 88.7)	79.6 (53.2, 96.4)	89.6 (65.8, 99.2)	38		16.8 (6.6, 39.2)	25.8 (10.7, 54.3)	36.3 (15.7, 69.7)	47.5 (21.5, 81.9)
B	0	292	59.0 (31.2, 88.1)	79.4 (49.0, 97.6)	88.3 (60.2, 99.3)	92.9 (67.8, 99.8)	96.4 (75.7, 100.0)	58	25.8 (11.8, 51.0)	41.1 (20.2, 71.2)	51.3 (26.6, 81.3)	58.8 (31.6, 87.4)	67.0 (37.8, 92.5)
	1	159		63.9 (24.5, 97.5)	80.8 (37.4, 99.7)	91.8 (50.4, 100.0)	97.2 (62.9, 100.0)	38		25.1 (7.4, 66.3)	37.5 (12.1, 82.0)	50.9 (17.6, 92.7)	63.7 (24.0, 97.6)
C	0	292	24.9 (19.0, 32.3)	39.9 (32.1, 48.8)	49.9 (41.2, 59.3)	57.3 (48.1, 66.9)	65.5 (56.0, 74.9)	58	9.2 (5.2, 15.7)	15.7 (9.3, 25.7)	20.7 (12.5, 33.0)	24.8 (15.2, 38.9)	30.0 (18.7, 45.9)
	1	159		33.4 (19.7, 53.0)	48.3 (30.7, 69.5)	63.2 (42.5, 83.5)	75.9 (54.5, 92.4)	38		10.9 (4.3, 26.2)	17.1 (7.1, 38.1)	24.7 (10.5, 51.7)	33.3 (14.6, 64.6)
D	0	292	18.9 (13.7, 25.8)	31.1 (23.6, 40.1)	39.7 (31.0, 49.7)	46.3 (36.8, 57.0)	54.1 (43.7, 65.3)	58	6.8 (3.7, 12.2)	11.7 (6.6, 20.2)	15.6 (9.0, 26.2)	18.8 (11.0, 31.1)	23.0 (13.6, 37.3)
	1	159		7.3 (3.6, 14.5)	11.6 (6.0, 21.7)	17.0 (9.1, 30.5)	23.3 (12.8, 40.3)	38		2.1 (0.8, 5.8)	3.4 (1.3, 8.9)	5.2 (2.0, 13.1)	7.3 (2.8, 18.0)

Factors in bold represent variation from Profile A.

*n* number of individuals, *CI* confidence interval, *yr* year.

Profile A: Variant group (truncating variants after aa781), mosaicism (absent), ever honeymoon period (yes), number of anti-seizure medication used in first year of life (0–3), age at seizure onset (>1.5 months), formal therapy during first year of life (yes).

Profile B: Variant group (truncating variants after aa781), **mosaicism (present)**, ever honeymoon period (yes), number of anti-seizure medication used in first year of life (0–3), age at seizure onset (>1.5 months), formal therapy during first year of life (yes).

Profile C: **Variant group (missense/in-frame variants within catalytic domain)**, mosaicism (absent), ever honeymoon period (yes), **number of anti-seizure medication used in first year of life (≥4)**, age at seizure onset (>1.5 months), formal therapy during first year of life (yes).

Profile D: **Variant group (truncating variants between aa172 and aa781)**, mosaicism (absent), **ever honeymoon period (no)**, **number of anti-seizure medication used in first year of life (≥4)**, **age at seizure onset (≤1.5 months)**, **formal therapy during first year of life (no)**.

**Table 4:** Estimated probability of independent walking at 1.5, 2, 4, and 6 years of age in individuals with CDKL5 Deficiency Disorder, by sex, profile of explanatory variables and starting age of observation.

Profile	Start age (yr)	Female (age in year)					Male (age in year)				
			Probability % (95% CI)					Probability % (95% CI)			
		*n*	1.5	2	4	6	*n*	1.5	2	4	6
A	0	268	6.8 (3.0, 14.8)	10.3 (5.0, 20.4)	30.4 (17.6, 49.3)	39.7 (23.9, 60.8)	57	5.2 (1.9, 13.6)	7.9 (3.1, 19.1)	24.0 (11.0, 47.7)	31.9 (15.2, 59.2)
	1	216		15.5 (6.5, 34.5)	49.7 (27.0, 77.6)	60.9 (35.2, 86.8)	46		12.1 (4.0, 33.9)	41.0 (16.9, 77.9)	51.4 (22.6, 87.0)
	1.5	198		5.1 (1.4, 18.6)	46.3 (23.4, 76.5)	59.3 (32.4, 87.3)	42		4.2 (0.9, 18.3)	39.4 (14.9, 78.9)	51.6 (21.1, 89.1)
B	0	268	16.1 (4.7, 47.6)	23.8 (7.5, 61.2)	50.7 (24.4, 94.7)	71.9 (32.5, 98.3)	57	12.5 (3.8, 36.8)	18.6 (6.1, 48.9)	49.8 (20.4, 87.5)	61.8 (27.4, 94.4)
	1	216		27.0 (6.8, 75.6)	72.2 (26.8, 99.5)	82.6(34.8, 99.9)	46		21.5 (5.6, 63.9)	62.7 (22.7, 97.7)	74.0 (29.8, 99.4)
	1.5	198		11.0 (1.9, 51.7)	74.7 (26.9, 99.8)	86.3 (36.6, 100.0)	42		9.0 (1.6, 41.7)	67.0 (24.4, 98.7)	79.9 (33.6, 99.8)
C	0	268	2.5 (1.1, 5.3)	3.8 (1.9, 7.5)	12.1 (6.9, 20.5)	16.4 (9.7, 27.6)	57	1.9 (0.7, 5.1)	2.9 (1.1, 7.3)	9.3 (4.0, 20.7)	12.7 (5.6, 27.3)
	1	216		4.5 (1.7, 12.0)	17.2 (7.5, 36.8)	22.8 (10.2, 46.4)	46		3.5 (1.0, 11.6)	13.5 (4.6, 36.3)	18.0 (6.3, 45.7)
	1.5	198		1.8 (0.5, 7.2)	19.6 (8.4, 41.8)	27.1 (12.1, 53.9)	42		1.5 (0.3, 7.1)	16.1 (5.3, 43.5)	22.4 (7.6, 55.7)
D	0	268	2.5 (1.1, 5.6)	3.9 (1.9, 7.9)	12.4 (6.9, 21.7)	16.9 (9.7, 28.6)	57	1.9 (0.7, 5.5)	3.0 (1.1, 7.9)	9.6 (3.9, 22.3)	13.1 (5.5, 29.4)
	1	216		1.5 (0.6, 4.2)	6.1 (2.6, 14.1)	8.2 (3.5, 18.5)	46		1.2 (0.3, 4.0)	4.7 (1.6, 13.8)	6.4 (2.1, 18.2)
	1.5	198		0.3 (0.1, 1.4)	3.7 (1.3, 10.1)	5.3 (1.9, 14.0)	42		0.3 (0.0, 1.4)	3.0 (0.8, 10.4)	4.3 (1.2, 14.5)

Factors in bold represent variation from Profile A.

*n* number of individuals, *CI* confidence interval, *yr* year.

Profile A: Variant group (truncating variants after aa781), mosaicism (absent), ever honeymoon period (yes), number of anti-seizure medication used in first year of life (0–3), age at seizure onset (>1.5 months).

Profile B: Variant group (truncating variants after aa781), **mosaicism (present)**, ever honeymoon period (yes), number of anti-seizure medication used in first year of life (0–3), age at seizure onset (>1.5 months).

Profile C: **Variant group (missense/in-frame variants within catalytic domain)**, mosaicism (absent), ever honeymoon period (yes), **number of anti-seizure medication used in first year of life (≥4)**, age at seizure onset (>1.5 months).

Profile D: **Variant group (truncating variants between aa172 and aa781)**, mosaicism (absent), **ever honeymoon period (no), number of anti-seizure medication used in first year of life (≥4), age at seizure onset (≤1.5 months)**.

Regarding independent walking, the estimated probability of achieving this milestone at 6 years of age when observed from birth was 39.7% (95% CI 23.9, 60.8) and 16.9% (95% CI 9.7, 28.6) for females with the most and least favourable profile, respectively (Table 4). The probability changed to 60.9% (95% CI 35.2, 86.8) and 8.2% (95% CI 3.5, 18.5) when additional explanatory variables were included in the modelling when observed from one year of age (Table 4). Presence of mosaicism appeared to expedite achievement of either milestone in females as well as males with the most favourable profile, as the likelihood of independent sitting and walking increased in individuals with Profile B, which was the same as Profile A except for the presence of mosaicism (Tables 3 and 4).

## Discussion

Despite being a severe DEE [11], considerable variability has been demonstrated in CDD with motor ability ranging from inability to maintain independent sitting to the ability to walk and run. Similar variation in hand function and communication has also been shown [11]. In this study we aimed to investigate how the age at acquisition of major milestones might be influenced by a range of factors early in life. We found the likelihood of children achieving independent sitting by the age of one year was 22% (median age three years), and in general children with late (after aa781) truncating variants or variants resulting in no functional protein were more likely to sit than those with truncating variants between aa172 and aa781. If a child had not achieved independent sitting by the age of one year, factors that increased their likelihood of subsequently achieving this skill were having a favourable genotype (in this case a truncating variant after aa781), having a later onset of seizures (after six weeks), having been on three or less ASMs in the first year of life, having experienced a honeymoon period and having received therapy in the first year of life. Females had a much greater likelihood than males of achieving independent sitting as had males where a mosaic genotype had been reported. With respect to walking the likelihood was also increased for those with a truncating variant after aa781 and according to similar factors as for sitting.

Prediction models have previously been used to assess the likelihood of an infant born preterm surviving to various ages without a major disability [19] and to estimate the likelihood of seizure onset in Rett syndrome [20]. They allow the clinician to relate these factors to their own particular patient in order to provide some degree of prognostic guidance. In the case of CDD we have shown that it is possible to make use of information both available at birth (i.e., gender, genotype and presence of mosaicism) and subsequently (i.e., age at seizure onset, use of ASMs, occurrence of a honeymoon period and engagement in therapy programs) to predict likely later development. In the future, our models could be further refined and extended to increase their utility as we collect more data on an even larger number of cases.

This study has both strengths and weaknesses. An obvious strength is the international data collection of 350 genetically confirmed cases accumulated over a period of nine years. This has been made possible because of ongoing support from advocacy associations and is currently the largest available dataset on this disorder. A further strength is that the questions we ask are important both to families and to the clinicians who provide counselling to them. One important limitation is that we had to use a broad classification of genotype because with over two hundred unique pathogenic variants represented in the ICDD the number that were recurrent was too small (with only 13/207 variants affecting three or more individuals) [7] to allow us to analyse according to individual variants. The latter much more preferable option would likely provide even better estimates given our previous findings of some statistical differences in development and in severity between some individual variants which did not necessary correspond to our finding here according to variant group. For instance, in our previous study those with a p.Arg134* had the lowest severity score and a relatively high developmental score but would not be categorised as a late truncating variant rather as one with “no functional protein.” Similarly, p.Arg550*, the variant with the highest developmental score and the next lowest severity score after p.Arg134*, was classified as a truncating variant occurring between amino acid (aa) 172 and aa781 and would not be categorised as a late truncating variant which one might have expected given the relationship we have found here with the late truncating group and earlier milestone achievement. However, including more recurrent variants which could be analysed individually would be dependent on a considerable increase in recruitment, which is challenging for an ultra-rare disorder such as CDD [9, 10]. With respect to mosaicism its presence may not have been reported uniformly by laboratories and it is possible that for some children this information may have been missed. A further limitation is the availability and choice of variables including the absence of detail on therapy interventions, accessibility to which may depend on geographical location and socioeconomic circumstances [21]. Furthermore, not all families were able to provide the specific information ideally required in relation to the honeymoon period. We improved the power and strength of the estimates of the model we presented in relation to walking by not restricting to children who were able to sit. However, in a supplementary analysis which included this restriction we were able to show, as might be expected, that the earlier the child achieved sitting the more likely they were to achieve walking (Supplementary Table 3). Regarding ASMs we cannot account for clinician variability in prescribing practices. Finally, while CDD is a rare disorder with considerable genetic heterogeneity, and, although our sample size is one of the largest available, the accuracy and precision of estimates involving specific combinations of factors could still be compromised.

The factors that we investigated were (1) those determined at birth i.e., gender, genotype group and presence of mosaicism, (2) those that occurred subsequently relating to epilepsy i.e., age of onset, number of ASMs and presence of a honeymoon period and (3) finally the presence of therapy intervention in the first year of life. It had previously been reported that males were considerably more impaired than females [11, 12]. We have now shown for the first time, but as might be expected, [22] that the presence of mosaicism, affecting seven (12%) of the 58 males, beneficially altered their developmental trajectory such that the overall likelihood of walking by age six years was not dissimilar in males and females. We could postulate that gene replacement therapy might also alter the phenotype by improving motor skills through a similar mechanism to mosaicism [23]. With respect to genotype our findings in relation to late truncating variants after aa781 mirrored those found previously using a much smaller sample [12]. However, in this study we also included the impact of early factors relating to epilepsy. For instance, if a female child aged 12 months with a late truncating variant had not learned to sit by the age of 12 months the likelihood that she would achieve sitting would increase from 47.7% at 1.5 years to 89.6% by the age of five years if their seizures started later, they had used fewer ASMs, and they had experienced a honeymoon period. We acknowledge that most of these factors are not modifiable and we do not know whether the honeymoon period is an inherent component of the natural history of this disorder or whether it relates to a specific management regime. We also know that the ASM regime may be a consequence of the epilepsy severity and may or may not be related to clinician prescribing habit.

In terms of physical therapies, the majority of children did receive therapy in the first year of life making it more difficult to use this as a discriminating factor. We can certainly postulate that in general early therapy has the potential to achieve more because of greater neuroplasticity [24]. However, in CDD therapy sessions may often be interrupted by episodes of epilepsy which if protracted can also result in regression of skills [17]. Moreover, we had limited detail on the quality and intensity of the intervention which could have considerable relevance. The data has been collected over a period of nine years with the oldest individuals being born two to three decades ago. Availability of intervention may have improved over time, but we do not know what the impact of an early confirmed CDD diagnosis is on the likelihood of institution of therapy.

In conclusion this paper is but a first attempt to understand the factors underlying the variability of motor development in CDD and to assist clinicians in providing prognostic guidance to their patients’ families. In the future aggregation of unique data from multiple settings could potentially further increase sample size and the range of variables that could be investigated. Most importantly it could provide the opportunity to increase the numbers of those with individual recurrent variants and thereby further clarify the role of genetic and environmental factors in attainment of motor milestones in this ultra-rare disorder.

## Supplementary information

Supplementary Table 1

Supplementary Table 2

Supplementary Table 3

## Data Availability

The dataset analysed during the current study is not publicly available but may be available from the corresponding author on reasonable request following an application to and with approval from the local ethics committee.
