# Correction to: Factors influencing the attainment of major motor milestones in CDKL5 deficiency disorder

**DOI:** 10.1038/s41431-022-01209-4

**Published:** 2022-10-13

**Authors:** Kingsley Wong, Mohammed Junaid, Scott Demarest, Jacinta Saldaris, Tim A. Benke, Eric D. Marsh, Jenny Downs, Helen Leonard

**Affiliations:** 1 Telethon Kids Institute, The University of Western Australia, Perth, WA Australia; 2 Children’s Hospital Colorado, Pediatric Neurology, University of Colorado School of Medicine, Aurora, USA; 3 Division of Neurology, Children’s Hospital of Philadelphia, School of Medicine, University of Pennsylvania, Philadelphia, USA

**Keywords:** Genetics research, Paediatrics

Correction to: *European Journal of Human Genetics* 10.1038/s41431-022-01163-1, published online 18 August 2022

In the sentence beginning ‘Similarly...’ in the third paragraph in Discussion, “p.Arg559*” should have been “p.Arg550*”.

The original article has been corrected.

