# Supplementary material for: Factors influencing the attainment of major motor milestones in CDKL5 deficiency disorder
Source: Eur J Hum Genet. 2022 Aug 18;31(2):169–78. doi: 10.1038/s41431-022-01163-1 (PMC9905550; doi:10.1038/s41431-022-01163-1)
Supplement: Supplementary file 1 — Supplementary Table 1 [file 41431_2022_1163_MOESM1_ESM.docx]

**Supplementary Table 1. Profile of explanatory variables**

| **Profile** | **A** | **B** | **C** | **D** |
| --- | --- | --- | --- | --- |
| Variant group | Truncating variants after aa781 | Truncating variants after aa781 | Missense/in-frame variants within catalytic domain | Truncating variants between aa172 and aa781 |
| Mosaicism | Absent | Present | Absent | Absent |
| Ever honeymoon period | Yes | Yes | Yes | No |
| Number of ASM used in first year of life | 0-3 | 0-3 | ≥ 4 | ≥4 |
| Age at seizure onset (month) | >1.5 months | >1.5 months | >1.5 months | ≤1.5 months |
| Formal therapy during first year of life^#^ | Yes | Yes | Yes | No |

aa, amino acid; ASM, antiseizure medication

^#^ Not considered for independent walking.
