# Supplementary material for: Factors influencing the attainment of major motor milestones in CDKL5 deficiency disorder
Source: Eur J Hum Genet. 2022 Aug 18;31(2):169–78. doi: 10.1038/s41431-022-01163-1 (PMC9905550; doi:10.1038/s41431-022-01163-1)
Supplement: Supplementary file 2 — Supplementary Table 2 [file 41431_2022_1163_MOESM2_ESM.docx]

**Supplementary Table 2. Summary of characteristics, independent sitting and walking among 350 individuals with CDKL5 Deficiency Disorder**

| **Characteristics** | n (%) or median (interquartile range; range) |
| --- | --- |
| **Sex** |  |
| Female | 292 (83.4) |
| Male | 58 (16.6) |
| **Age (year)** | 5.7 (2.4,10.9; 0.3-35.0) |
| <1 | 36 (10.3) |
| 1 to 4 | 126 (36.0) |
| 5 to 10 | 102 (29.1) |
| 11 or above | 86 (24.6) |
| **Variant group** |  |
| No functional protein | 87 (24.9) |
| Missense/in-frame variants within catalytic domain | 111 (31.7) |
| Truncating variants between aa172 and aa781 | 96 (27.4) |
| Truncating variants after aa781 | 37 (10.6) |
| Other variants | 19 (5.4) |
| **Mosaicism** |  |
| Present | 10 (2.9) |
| Absent | 340 (97.1) |
| **Age at seizure onset (month)** |  |
| ≤1.5 | 198 (56.6) |
| >1.5 | 137 (39.1) |
| Missing | 15 (4.3) |
| **Number of anti-seizure medications used in first year of life** |  |
| Zero | 15 (4.3) |
| One | 22 (6.3) |
| Two | 62 (17.7) |
| Three | 60 (17.1) |
| Four | 55 (15.7) |
| Five or more | 112 (32.0) |
| Missing | 24 (6.9) |
| **Ever honeymoon period** |  |
| Yes | 197 (56.3) |
| No | 136 (38.9) |
| Missing | 17 (4.9) |
| **Formal therapy during first year of life** |  |
| Yes | 222 (63.4) |
| No | 90 (25.7) |
| Missing | 38 (10.9) |
| **Independent sitting** |  |
| Yes | 177 (50.6) |
| No | 173 (49.4) |
| **Independent walking^^^** |  |
| Yes | 57 (16.3) |
| No | 268 (76.6) |
| Missing | 25 (7.1) |

^^^Excludes individuals with missing data on time to walking.
