# Supplementary material for: Factors influencing the attainment of major motor milestones in CDKL5 deficiency disorder
Source: Eur J Hum Genet. 2022 Aug 18;31(2):169–78. doi: 10.1038/s41431-022-01163-1 (PMC9905550; doi:10.1038/s41431-022-01163-1)
Supplement: Supplementary file 3 — Supplementary Table 3 [file 41431_2022_1163_MOESM3_ESM.docx]

**Supplementary Table 3** **Multivariable regression analysis of time to independent walking conditional to independent sitting in individuals with CDKL5 Deficiency Disorder, by starting age of observation**

| **Starting age of observation (year)** | **0** | **1** | **1.5** | **0** | **1** | **1.5** |
| --- | --- | --- | --- | --- | --- | --- |
| **n** | 174 | 151 | 139 | 174 | 151 | 139 |
|  | HR (95% CI) | | | β (95% CI) | | |
| **Sex** |  |  |  |  |  |  |
| Female | 0.48 (0.21, 1.07) | 0.36 (0.13, 1.03) | 0.36 (0.12, 1.12) | -0.74 (-1.55, 0.71) | -1.02 (-2.08, 0.03) | -1.02 (-2.16, 0.11) |
| Male | Ref | Ref | Ref | Ref | Ref | Ref |
| **Variant group** |  |  |  |  |  |  |
| Truncating variants between aa178 and aa781 | Ref | Ref | Ref | Ref | Ref | Ref |
| Truncating variants after aa781 | 2.18 (0.94, 5.02) | 1.23 (0.50, 3.01) | 1.47 (0.53,4.02) | 0.78 (-0.06,1.61) | 0.21 (-0.69, 1.11) | 0.38 (-0.63, 1.39) |
| No functional protein | 1.02 (0.47, 2.23) | 0.69 (0.30, 1.57) | 0.70 (0.27, 1.83) | 0.02 (-0.76, 0.80) | -0.38 (-1.20, 0.45) | -0.36 (--1.32, 0.60) |
| Missense/in-frame | 0.63 (0.28, 1.47) | 0.47 (0.18, 1.25) | 0.68 (0.23, 1.97) | -0.45 (-1.29, 0.39) | -0.75 (-1.71, 0.23) | -0.39 (-1.46, 0.68) |
| Other variants | 1.51 (0.49, 4.63) | 0.71 (0.18, 2.80) | 0.74 (0.16, 3.45) | 0.41 (-0.70, 1.53) | -0.34 (-1.70, 1.03) | -0.31 (-1.85, 1.24) |
| **Mosaicism** |  |  |  |  |  |  |
| Absent | Ref | Ref | Ref | Ref | Ref | Ref |
| Present | 2.51 (0.89, 7.08) | 1.42 (0.38, 5.30) | 1.70 (0.44, 6.64) | 0.36 (-0.71, 1.44) | 0.35 (-0.96, 1.67) | 0.53 (-0.83, 1.89) |
| **Ever honeymoon period** |  |  |  |  |  |  |
| No | - | Ref | Ref | - | Ref | Ref |
| Yes | - | 1.66 (0.87, 3.17) | 2.27 (1.09, 4.71) | - | 0.51 (-0.14, 1.15) | 0.82 (0.09,1.55) |
| **Number of ASM used in first year of life** |  |  |  |  |  |  |
| 0-3 | - | 0.99 (0.52, 1.91) | 0.99 (0.48, 2.04) | - | -0.006 (-0.66, 0.65) | -0.01 (-0.74,0.71) |
| ≥4 | - | Ref | Ref | - | Ref | Ref |
| **Age at seizure onset (month)** |  |  |  |  |  |  |
| ≤1.5 | - | Ref | Ref | - | Ref | Ref |
| >1.5 | - | 1.45 (0.77, 2.70) | 1.45 (0.73, 2.90) | - | 0.37 (-0.26, 1.00) | 0.37 (-0.32, 1.07) |
| **Age at independent sitting (years)** | - | 0.43 (0.28, 0.66) | 0.47 (0.30, 0.73) |  | -0.85 (-1.27, -0.42) | -0.75 (-1.19,-0.31) |
